# The relationship between digital health literacy, self-efficacy, and self-management behaviors in patients with diabetes-related foot disease: a cross-sectional study

**DOI:** 10.3389/fpubh.2026.1850957

**Published:** 2026-06-24

**Authors:** Jianxia Yao, Yongxin Huang, Jinqi Huang, Qiqi Yao, Shuping Li, Shanshan Wu, Aiqiong Huang, Baogen Xie, Ruilin Li, Qun Chen, Min Cai

**Affiliations:** 1 Department of Burn and Plastic Surgery, The First Hospital of Putian City, Putian, Fujian, China; 2 Department of Interventional Vascular Surgery, The First Hospital of Putian City, Putian, Fujian, China; 3 Department of Endocrinology, The First Hospital of Putian City, Putian, Fujian, China; 4 Department of Nutrition, The First Hospital of Putian City, Putian, Fujian, China; 5 Department of Psychology, The First Hospital of Putian City, Putian, Fujian, China; 6 Department of Cardiology, The First Hospital of Putian City, Putian, Fujian, China

**Keywords:** cross-sectional studies, diabetes-related foot disease, health literacy, patient self-management, self efficacy

## Abstract

**Objective:**

This study aimed to explore the relationship among digital health literacy, self-efficacy, and self-management behaviors in patients with diabetes-related foot disease (DRFD), and to analyze the mediating role of self-efficacy.

**Methods:**

A cross-sectional survey was conducted from February 2024 to December 2025 among 328 patients with DRFD in a endocrinology department of tertiary hospital. Participants were recruited using a consecutive sampling method and completed a general information questionnaire, the eHealth Literacy Scale (eHEALS), the Diabetes Management Self-Efficacy Scale (DMSES), and the Diabetic Foot Self-Management Behavior Scale (DFSMBS). Mediation analysis was performed using the SPSS Process Macro (Model 4), with bootstrap resampling (5,000 iterations) to calculate 95% confidence intervals. The study was conducted in accordance with the STROBE guidelines.

**Results:**

Digital health literacy was moderately positively correlated with self-management behaviors (*r* = 0.42, *p* < 0.01). Self-efficacy was strongly positively correlated with self-management behaviors (*r* = 0.53, *p* < 0.01). These associations remained significant after controlling for potential confounding variables (including age, disease duration, and Wagner grade). Furthermore, self-efficacy partially mediated the relationship between digital health literacy and self-management behaviors, with a mediation effect of 0.23 [95% CI (0.156, 0.328)], accounting for 41.8% of the total effect.

**Conclusion:**

Improving digital health literacy in patients with DRFD is directly associated with better self-management behaviors and is indirectly associated with them through increased self-efficacy. Clinical practice should integrate digital health education with psychological empowerment strategies to optimize self-management.

## Graphical abstract

**Figure:**
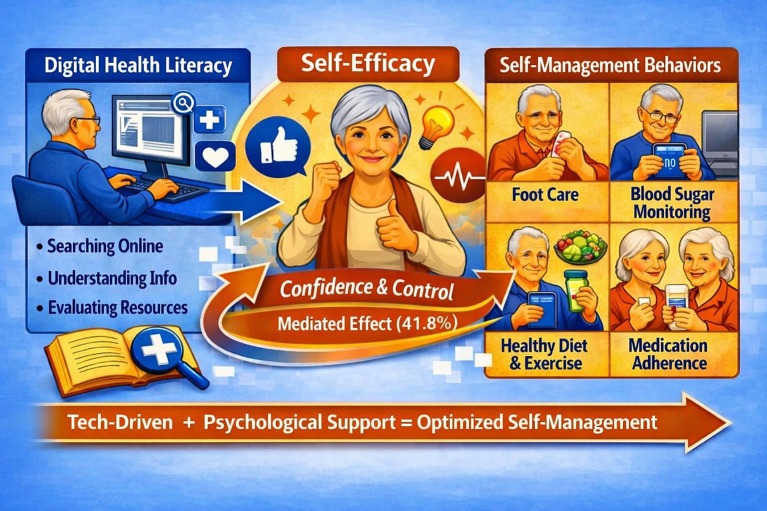
The graphical abstract illustrates the mediating role of self-efficacy in the relationship between digital health literacy and self-management behaviors among patients with DRFD. Digital health literacy provides access to and understanding of health information, while self-efficacy enhances confidence and perceived control, thereby facilitating the transformation of knowledge into concrete self-care practices such as foot care, blood glucose monitoring, healthy diet and exercise, and medication adherence. The model highlights that digital health literacy not only exerts a direct effect on self-management behaviors but also indirectly promotes them through self-efficacy, accounting for 41.8% of the total effect. This integrated pathway underscores the importance of combining technology-driven interventions with psychological support and health education to optimize patient outcomes.

## Introduction

Diabetes-related foot disease (DRFD) is one of the most severe complications of diabetes, characterized by foot ulcers, infections, and tissue necrosis, which significantly impaires quality of life and impose a heavy socioeconomic burden (1). According to the International Diabetes Federation (IDF) Diabetes Atlas 11th edition (2025), the global prevalence of diabetes has reached 11.1%, affecting approximately 589 million adults, and is projected to rise to 13% by 2050, impacting 853 million people (2). The lifetime risk of DRFD is 19–34%, with about 20% of patients eventually requiring lower-limb amputation (3). Alarmingly, the 5-year mortality rate after major amputation with diabetes-related foot ulcer (DRFU) is 50–70%, exceeding that of many malignant tumors (4). Thus, DRFD management has become a pressing global public health challenge.

Effective self-management behaviors, such as daily foot inspection, wound care, blood glucose monitoring, use of offloading devices, and lifestyle modifications—are central to DRFD management (5). Evidence shows that adherence to these behaviors reduces ulcer recurrence, hospitalization, and amputation risk, while improving glycemic control and quality of life (6). However, many patients struggle with self-management due to neuropathy, vascular disease, mobility limitations, pain, psychological burden, and limited access to health information. Studies indicate that 50–70% of patients fail to perform daily foot checks, with even lower adherence in resource-limited settings (7, 8). In underdeveloped regions, this problem is even more pronounced, with studies showing that only 12.2% of patients in India demonstrate adequate foot care practices (9). Therefore, enhancing self-management capacity among patients with DRFD has become a critical task in clinical nursing and public health interventions.

In the rapidly evolving era of “Internet + Healthcare,” digital health technologies provide innovative solutions to overcome the bottlenecks in self-management of DRFD. Compared with other diabetic populations, patients with DRFD face higher risks of ulcer recurrence and disability, making digital health literacy essential for timely access to reliable information and adherence to foot care (10). Digital health literacy refers to an individual’s ability to access, understand, evaluate, and apply health information using digital technologies such as the internet, mobile applications, wearable devices, remote monitoring systems, and AI-assisted tools (11). For patients with DRFD, digital health literacy enables real-time monitoring of blood glucose fluctuations via smartphone applications, access to professional foot care guidance through telemedicine platforms, and peer support and experience sharing in online communities, thereby achieving personalized, precise, and continuous disease management (12). Previous studies have confirmed that digital intelligent interventions (including WeChat platforms, mobile applications, and remote monitoring) can significantly improve self-management behaviors in patients with DRFD, with 94% of studies reporting positive outcomes (13). This suggests that digital health literacy is a key factor in breaking through the barriers of self-management in DRFD.

Digital health literacy provides patients with tools and channels, but whether it can be transformed into sustained health behaviors depends on self-efficacy, a critical psychological factor. According to Bandura’s social cognitive theory, self-efficacy is the core construct that explains how individuals translate knowledge into behavior. Self-efficacy refers to an individual’s confidence and belief in their ability to perform specific behaviors and achieve expected outcomes (14). In chronic diseases, self-efficacy serves as the psychological bridge linking digital health literacy (knowledge acquisition) with self-management behaviors (behavioral outcomes) (15). Among patients with DRFD, self-efficacy has been shown to be positively associated with foot care adherence, ulcer healing, pain control, and psychological well-being (16). Furthermore, studies have found that the use of digital health technologies (“Internet +,” smartphone applications) can indirectly influence foot care behaviors through the mediating role of self-efficacy (17). Guided by social cognitive theory, this study focuses on examining the mediating effect of self-efficacy in the relationship between digital health literacy and self-management behaviors in patients with DRFD, aiming to reveal the psychological mechanisms underlying digital health interventions.

**Figure 1:**
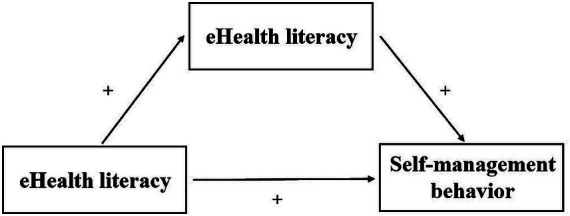
Hypothesized mediation model. The hypothesized model illustrates the proposed relationships among digital health literacy, self-efficacy, and self-management behaviors in patients with DRFD. Arrows indicate the assumed directional associations between variables.

Based on this rationale, we constructed a mediation model (Figure 1) to elucidate pathways linking digital health literacy, self-efficacy, and self-management, thereby providing evidence for precision interventions in DRFD care.

## Methods

### Study design and participants

A cross-sectional study was conducted using consecutive sampling. A total of 328 patients with diabetic foot who attended the endocrinology department of the First Hospital of Putian City between February 2024 and December 2025 were recruited. Inclusion criteria: (a) Met the diagnostic criteria of the Guidelines for the Diagnosis and Treatment of DRFD and confirmed as diabetic foot patients; (b) Age ≥18 years; (c) Stable condition without acute cardiovascular or cerebrovascular events or severe infection; (d) Ability to use smartphones or other digital devices with basic communication skills; (e) Voluntary participation with signed informed consent. Exclusion criteria: (a) Severe psychiatric disorders or cognitive impairment preventing completion of the questionnaire; (b) Severe hepatic or renal failure or malignant tumors with limited life expectancy; (c) Recent major surgery (within 3 months) or acute complications; (d) Inability to independently complete questionnaires or follow-up (e.g., visual or language impairment); (e) Refusal to participate or withdrawal during the study. This study was conducted and reported in accordance with the STROBE guidelines.

### Sample size calculation

According to the sample size estimation method for mediation analysis, with a significance level set at *α* = 0.05 and statistical power at 0.80, assuming a correlation coefficient of approximately 0.40 between the independent variable and the mediator (18), the required sample size was calculated to be about 280 cases. Considering an estimated 15% of invalid or missing questionnaires, the final sample size was determined to be 322 participants.

### Study instruments

#### General information questionnaire

The general information questionnaire was developed by the research team based on a literature review and included variables such as age, sex, disease duration, Wagner classification, educational level, medical history, and digital device usage.

#### eHealth literacy scale (eHEALS)

Digital health literacy was measured using the validated Chinese version of eHEALS. The original scale was developed by Norman and Skinner (11) to assess the ability to access, understand, evaluate, and apply online health information. The Chinese version was translated and validated by Chen et al. (19), consisting of 8 items on a 5-point Likert scale (total score 8–40). Higher scores indicate better literacy. It has been widely applied among older adults and diabetic populations in China (20), with Cronbach’s *α* ranging from 0.87–0.91. In this study, Cronbach’s *α* was 0.92.

#### Diabetes management self-efficacy scale (DMSES)

Patients’ self-efficacy levels were assessed using the Chinese version of DMSES. originally developed based on Bandura’s social cognitive theory (21). The Chinese version was validated by Li et al. (22) and has been widely used among diabetic populations. Reported Cronbach’s *α* ranges from 0.611–0.947. In this study, Cronbach’s *α* was 0.89.

#### Diabetic foot self-management behavior scale (DFSMBS)

Patients’ self-management behaviors were measured using the DFSMBS, developed by domestic scholars based on the diabetes self-management framework (23). It covers disease monitoring and control management, daily life management, and foot care, with 16 items on a 4-point Likert scale. Previous studies reported Cronbach’s *α* of 0.83–0.85 (24). In this study, Cronbach’s *α* was 0.83.

Additionally, the Chinese versions of the study instruments were translated and culturally adapted following standard procedures. Content validity was confirmed through expert review and pilot testing. Reliability was assessed in the current sample, and higher scores consistently indicate better outcomes across all instruments. Permission for the use of all study instruments was obtained from the original developers or copyright holders.

### Data collection and quality control

The questionnaires were distributed on-site by trained research staff in both paper and electronic formats, with a completion time of approximately 20–30 min. Participants with adequate literacy completed the questionnaires independently, while older participants or those with limited literacy received interviewer assistance to ensure accuracy. To ensure consistency, standardized administration procedures were applied across both paper-based and electronic formats, and subsequent comparison revealed no significant differences in responses. Upon completion, the questionnaires were immediately collected and checked for completeness. To ensure data quality, all investigators received standardized training prior to data collection, becoming familiar with the scale content and survey procedures. During the survey, researchers provided necessary explanations to avoid errors caused by misunderstanding. After collection, questionnaires were examined for completeness and logical consistency, and those with substantial missing data (>10%) or patterned responses were excluded. Data entry was performed independently by two researchers with subsequent cross-checking to ensure accuracy. Random sampling and follow-up verification were conducted to further confirm the authenticity and reliability of the data.

### Data analysis

All data were analyzed using SPSS version 26.0. Descriptive statistics were used to summarize demographic and clinical characteristics: continuous variables were expressed as mean ± standard deviation (M ± SD), and categorical variables as frequencies and percentages. Pearson correlation analysis was applied to explore relationships among variables. Mediation effects were tested using the SPSS Process macro (Model 4), with age, disease duration, and Wagner classification included as covariates. The significance of mediation effects was assessed using the bootstrap method with 5,000 resamples to calculate 95% confidence intervals (CI); mediation was considered significant if the CI did not include zero. All statistical tests were two-sided, with *p* < 0.05 considered statistically significant.

## Results

### Demographic characteristics

A total of 328 patients with diabetic foot were enrolled using consecutive sampling, and all participants completed valid questionnaires, yielding a 100% effective response rate. The study population was predominantly middle-aged and older adults, with males accounting for 57.6%. Educational attainment was generally low, with 28.7% having primary school or below and 42.7% having junior high school, totaling 71.4% at junior high or below. The duration of diabetes was mainly 6–10 years (41.8%). Overall, the disease condition was relatively stable, with 83.9% of patients classified as Wagner grade 1–2. Digital device usage reached 78.4%, but only 32.6% of patients reported regularly using digital devices to obtain health information. Demographic, clinical characteristics, and digital device usage are presented in Table 1.

**Table 1:** Demographic and clinical characteristics of the study participants (*n* = 328).

Variables	Categories	Case (*n*)	Percentage (%)
Gender	Male	189	57.6
	Female	139	42.4
Age (years)	≤60	148	45.1
	61–70	123	37.5
	>70	57	17.4
Educational level	Primary school and below	94	28.7
	Junior high school	140	42.7
	Senior high school	66	20.1
	College and above	28	8.5
Course of diabetes (years)	≤5	100	30.5
	6–10	137	41.8
	>10	91	27.7
Wagner grade	Grade 1	155	47.3
	Grade 2	120	36.6
	Grade 3 and above	53	16.1
Marital status	Married	300	91.5
	Unmarried/divorced/widowed	28	8.5
Complicated with underlying diseases	Yes	273	83.2
	No	55	16.8
Digital device usage	Used	257	78.4
	Not used	71	21.6
Use of digital devices for health information acquisition	Yes	107	32.6
	No	221	67.4
Daily usage time of digital devices (h)	<1	91	35.4
	1–3	129	50.2
	>3	37	14.4

### Scores and correlation analysis of variables

As shown in Table 2, the mean scores were: digital health literacy (28.4 ± 6.2), self-efficacy (46.8 ± 6.9), and self-management behaviors (76.5 ± 8.4). Pearson correlation analysis revealed significant positive associations: digital health literacy with self-management behaviors (*r* = 0.42, *p* < 0.01); self-efficacy with self-management behaviors (*r* = 0.53, *p* < 0.01); and digital health literacy with self-efficacy (*r* = 0.38, *p* < 0.01).

**Table 2:** Scores and correlation analysis of variables (*n* = 328).

Variables	eHealth literacy	Self-efficacy	Self-management behavior	Score (M ± SD)
eHealth literacy	1	0.38^*^	0.42^*^	28.4 ± 6.2
Self-efficacy	0.38^*^	1	0.53^*^	6.8 ± 1.9
Self-management behavior	0.42^*^	0.53^*^	1	6.5 ± 1.4
Cronbach’s *α*	0.92	0.89	0.83	

Cronbach’s *α* indicates internal consistency reliability. ^*^p < 0.01, statistically significant (two-tailed test).

### Mediating effect test

**Figure 2:**
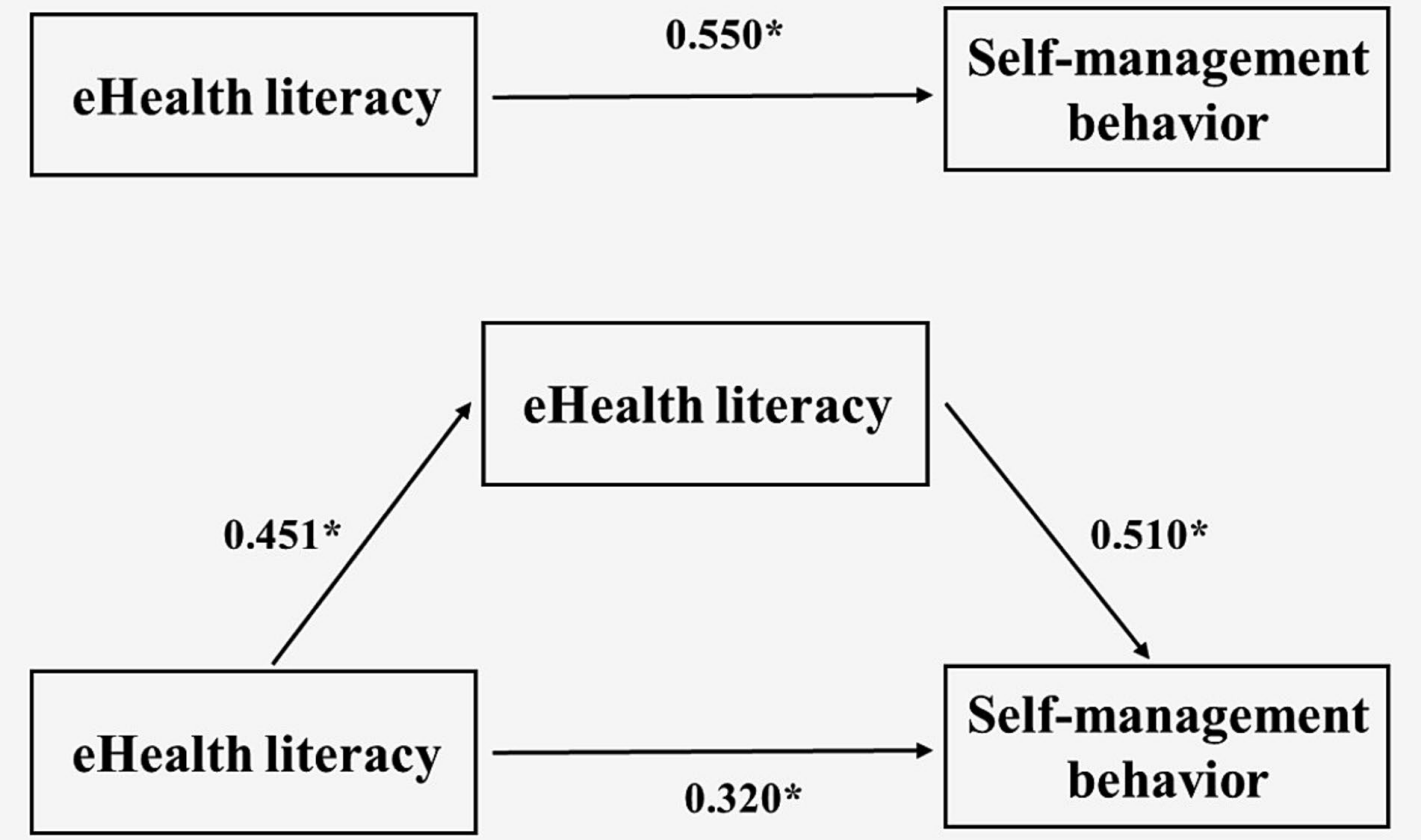
Mediating analysis of self-efficacy between digital health literacy and self-management behaviors. Digital health literacy was positively associated with self-efficacy (*β* = 0.451) and self-management behaviors (*β* = 0.320). Self-efficacy was positively associated with self-management behaviors (*β* = 0.510). The total effect of digital health literacy on self-management behaviors was *β* = 0.550. *β* represents standardized regression coefficients. ^*^p < 0.001 indicates statistical significance (two-tailed test).

As shown in Table 3, Figure 2, digital health literacy was positively associated with self-efficacy [*β* = 0.451, *p* < 0.001, 95% CI (0.322, 0.580)], and self-efficacy was positively associated with self-management behaviors [*β* = 0.510, *p* < 0.001, 95% CI (0.390, 0.630)]. After controlling for covariates, digital health literacy remained directly associated with self-management behaviors [*β* = 0.320, *p* < 0.001, 95% CI (0.192, 0.451)], accounting for 58.2% of the total effect. Meanwhile, self-efficacy played a partial mediating role between digital health literacy and self-management behaviors, with a mediation effect value of 0.23 [95% CI (0.156, 0.328)], accounting for 41.8% of the total effect. The overall effect was *β* = 0.550 [*p* < 0.001, 95% CI (0.433, 0.679)]. These findings suggest that self-efficacy serves as an important bridge between digital health literacy and self-management behaviors, indicating that digital health literacy is associated with improved self-management both directly and indirectly through self-efficacy.

**Table 3:** Mediating analysis of self-efficacy between eHealth literacy and self-management behaviors in patients with DRFD (*n* = 328).

Effect type	Path	*β*	*p*	95%CI	Mediating effect ratio (%)
Direct path-1	eHealth literacy → self-efficacy	0.451	<0.001*	0.322, 0.580	–
Direct path-2	Self-efficacy → self-management behavior	0.510	<0.001*	0.390, 0.630	–
Direct effect	eHealth literacy → self-management behavior	0.320	<0.001*	0.192, 0.451	58.2
Mediating effect	eHealth literacy → self-efficacy → self-management behavior	/	<0.001*	0.156, 0.328	41.8
Total effect	eHealth literacy → self-management behavior	0.550	<0.001*	0.433, 0.679	–

*β* represents standardized regression coefficients. *CI*, confidence interval. Mediation effect ratio (%) indicates the proportion of the total effect explained by the indirect path. *p<0.001, statistically significant (two-tailed test).

## Discussion

The novelty of this study lies in the systematic examination of the pathway linking digital health literacy, self-efficacy, and self-management behaviors among patients with DRFD, and in revealing the mediating mechanism of self-efficacy. Previous research has primarily focused on the impact of single factors on self-management, whereas this study, through mediation effect modeling, demonstrates that digital health literacy not only directly is associated with patients’ self-management behaviors but also indirectly is associated with improvements through self-efficacy. This finding provided new evidence for the psychological mechanisms underlying digital health interventions and enriches the application framework of social cognitive theory in DRFD management.

The results of this study showed a significant positive correlation between digital health literacy and self-management behaviors, which is consistent with Normand et al.’s (11) eHealth literacy theory and aligns with Zhou et al.’s (13) review on the effectiveness of digital interventions in improving self-management among patients with DRFD. However, this correlation is not a simple linear relationship between knowledge and behavior; rather, it reflected the dual role of digital health literacy in DRFD management. On the one hand, it provided patients with access to and understanding of health information; on the other hand, by enhancing patients’ ability to evaluate and apply information, it facilitates the transformation of knowledge into concrete care behaviors (25). In other words, digital health literacy served not only as an “information gateway” but also as a “prerequisite for behavioral transformation.” Furthermore, this study found that self-efficacy plays a partial mediating role, accounting for 41.8% of the total effect. This indicates that improvements in digital health literacy do not automatically translate into behavioral changes; whether patients take action depends on their belief in their own abilities. This finding is highly consistent with the “health literacy–self-efficacy–self-management” pathway proposed by Chen et al. (15) in chronic disease populations. Previous studies have also demonstrated that self-efficacy is closely associated with foot care adherence, ulcer healing speed, and psychological well-being in patients with DRFD (26), further supporting our conclusions. Taken together, these findings suggested that digital health literacy provides external resources, while self-efficacy functions as internal motivation. Their interaction determines whether patients can establish stable self-management behaviors.

Beyond theoretical contributions, these findings have practical implications for clinical care. Previous research has indicated that digital health tools are generally effective among patients with DRFD; however, without psychological support, their long-term adherence remains limited (12). Our results suggested that when promoting digital health tools in clinical practice, motivational interviewing, peer education, and psychological support should be implemented simultaneously to enhance patients’ self-efficacy, thereby achieving sustained improvements in self-management behaviors (27). This implied that healthcare professionals should not only teach patients how to use digital tools but also help them build confidence and a sense of capability in order to facilitate true behavioral transformation. Future intervention models should shift from a purely “technology-driven” approach to an integrated pathway combining “technology + psychology + education.” For example, nurses may strengthen patients’ behavioral beliefs through individualized motivational interviewing, foster a sense of belonging and continuity via peer support groups, and provide evidence-based educational materials to help patients understand and apply digital health information (16). More importantly, such comprehensive intervention models help mitigate inequalities arising from the digital divide, particularly among older patients or those with lower educational levels. Previous studies have demonstrated that simple, user-friendly digital tools, when combined with continuous psychological support, significantly enhance adherence and improve quality of life (28). This study not only offers new perspectives for individualized nursing care in patients with DRFD but also provides practical support for the transformation of chronic disease management models at the public health level.

This study revealed the mediating role of self-efficacy, suggesting that future intervention strategies should not only focus on the dissemination and application of technology but also integrate psychological support and behavioral motivation to achieve sustained improvements in health behaviors. In nursing practice, this means that when providing health education for patients with DRFD, nurses should combine the use of digital tools with the cultivation of patients’ self-efficacy. For older patients or those with lower educational levels, interventions should emphasize the design of simple and user-friendly digital health tools, accompanied by continuous guidance and feedback, to reduce inequalities caused by the digital divide. Such integrated strategies may help reduce health inequalities and improve long-term outcomes in DRFD management.

### Strengthens and limitations

The strength of this study lies in the systematic examination of the mediating mechanism linking digital health literacy, self-efficacy, and self-management behaviors in patients with DRFD, thereby enriching the theoretical framework of digital health and chronic disease management. However, several limitations should be acknowledged. First, the cross-sectional design does not allow for causal inference. Second, the sample was drawn from a single center, which may limit the generalizability of the findings. Third, questionnaire-based surveys are subject to social desirability bias. Fourth, the reliance on self-reported measures may introduce response bias, and because all variables were collected at the same time point using questionnaires, common method variance cannot be ruled out. Future studies should employ longitudinal follow-up or randomized controlled trials to further verify causal pathways and explore differences across populations, such as those in rural areas or older patients.

## Conclusion

This study revealed the pathway linking digital health literacy, self-efficacy, and self-management behaviors in patients with DRFD. The results demonstrated that digital health literacy not only directly promotes self-management behaviors but also exerts a partial mediating effect through self-efficacy. Clinical nursing practice should therefore aim to simultaneously enhance patients’ digital health literacy and self-efficacy during health education, with particular attention to older individuals and those with lower educational levels. Designing simple, user-friendly digital tools and providing continuous support are essential to achieving behavioral transformation. Future research should employ longitudinal or randomized controlled designs to further establish causal relationships and to develop differentiated intervention strategies.

## Data Availability

The data that support the findings of this study are available from the corresponding author upon reasonable request.
